# Pro-Inflammatory Cytokine Levels in HIV Infected and Uninfected Pregnant Women with and without Preeclampsia

**DOI:** 10.1371/journal.pone.0170063

**Published:** 2017-01-17

**Authors:** Niren Ray Maharaj, Alisa Phulukdaree, Savania Nagiah, Prithiksha Ramkaran, Charlette Tiloke, Anil Amichund Chuturgoon

**Affiliations:** 1 Department of Obstetrics and Gynaecology, Prince Mshiyeni Memorial Hospital, KwaZulu-Natal, South Africa; 2 Discipline of Medical Biochemistry, College of Health Sciences, University of KwaZulu-Natal, South Africa; Stellenbosch University, SOUTH AFRICA

## Abstract

**Introduction:**

Preeclampsia and HIV/AIDS are inflammatory conditions that contribute significantly to adverse maternal and foetal outcomes. The immune reconstitution effects of HAART on inflammatory mediators has not been adequately studied in pregnancy and may impact on the inflammatory cytokine network in women with co-morbid preeclampsia. Our study evaluated changes in pro-inflammatory cytokines IL-2, TNF-α, IFN-γ and IL-6 in HIV infected preeclamptic women on HAART.

**Methods:**

A prospective experimental study was conducted at Prince Mshiyeni Memorial Hospital between July 2013 and September 2014. One hundred and ninety three pregnant women were recruited into 4 groups: uninfected normotensive (50; 26%), infected normotensive (45; 23%), uninfected preeclamptic (53; 28%) and infected preeclamptic women (45; 23%). Serum levels of cytokines TNF-α, IFN- γ, IL-2 and IL-6 were determined using commercially available kits and a Cytometric Bead Array (CBA). Comparative data was recorded and analysed descriptively.

**Results:**

In the control groups (normotensive), significantly lower values were found in IL-2 (p = 0.010), TNF-α (p = 0.045), and IL-6 (p = 0.005); and a non-significant decrease was observed in IFN-γ (p = 0.345) in HIV infected women on HAART compared to uninfected controls. In the experimental group (preeclamptic) women, significantly reduced levels were observed in IL-2 and TNF-α (p = 0.001; p = 0.000) and non-significant decreases were observed in IFN-γ and IL-6 (p = 0.023; p = 0.086) in HIV infected women on HAART compared with uninfected preeclamptic women. Non-significant differences were observed between uninfected preeclamptic and normotensive women.

**Conclusion:**

In uncomplicated/normotensive pregnancies, HIV/HAART is associated with significant decreases in IL-2, TNF-α and IL-6, and in preeclamptic women significant decreases in IL-2 and TNF-α were observed. These findings suggest that HIV/HAART impacts on pro-inflammatory cytokines in women with co-morbid preeclampsia. This provides a platform for further research on immune reconstitution effects of HAART during pregnancy, and the development of potential immune modulation therapies for the management of preeclampsia.

## Introduction

Preeclampsia (PE), a multi-organ hypertensive disorder of pregnancy, and Human immunodeficiency virus/Acquired immunodeficiency syndrome (HIV/AIDS) are associated with significant maternal and perinatal morbidity and mortality, especially in poor resourced countries [1–3]. The treatment of PE still remains empiric and resolution is achieved by delivery [4]. Highly active antiretroviral therapy (HAART) has been shown to successfully reduce plasma HIV-1 viral load and vertical transmission rates in pregnancy [5, 6], and is now integrated with the management of HIV infection in pregnancy [7].

There is no consensus on the relationship between these conditions since data on the impact of HIV on the rate of PE are conflicting [8]. HAART may influence the postulated effect of HIV on the development of hypertensive disorders during pregnancy [9]. Although the underlying immunological changes in PE and HIV is not completely understood, it is generally accepted that both conditions are associated with inflammation [10, 11]. Inflammation is mediated by a variety of soluble factors, including a group of secreted polypeptides known as cytokines [12].

Cytokines can be classified as pro and anti-inflammatory and are considered important initiators and mediators of inflammation and endothelial dysfunction [13]. Cytokines such as interferon gamma (IFN-γ) and tumour necrosis factor alpha (TNF-α) are characteristic of T helper 1 (Th1) type immunity and mediate several cell mediated cytotoxic and inflammatory reactions [14, 15]. Interleukin (IL)-2 plays a critical role in regulating cellular and humoral chronic inflammatory responses; whilst IL-6, although possessing some anti-inflammatory properties, is observed in many chronic inflammatory and autoimmune disorders and serves as a marker for the systemic activation of pro-inflammatory cytokines [12, 16].

Preeclampsia is associated with a generalized systemic inflammatory response and subsequent release of pro-inflammatory cytokines that may trigger the maternal disease [17]. An increase in the IL-2/IL-4 and IFN-γ /IL-4 ratios, as well as elevated circulating levels of IL-6 and TNF-α have been reported in previous studies, suggesting a pro-inflammatory systemic environment in PE when compared to normal pregnancy [18].

During the progression of HIV infection, a Th1 (pro-inflammatory) to Th2 (eosinophilic/anti-inflammatory) cytokine shift has been observed, which appears to be counteracted with the usage of HAART [19]. Data on the immune reconstitution effects of HAART on pro-inflammatory cytokines in HIV infected women with PE is lacking. To further understand the effects of HAART on the inflammatory cytokine network in HIV infected PE women, we investigated IL-2, TNF-α, IFN-γ and IL-6 in women during the third trimester of pregnancy.

## Materials and Methods

### Study population and sample collection

Institutional ethical and hospital regulatory permission was obtained for the study (Biomedical Research Ethics Committee, University of KwaZulu-Natal, South Africa; reference number BE 119/11). After written consent was obtained, participants were recruited over a 14-month period from July 2013 to September 2014 from the maternity unit at Prince Mshiyeni Memorial Hospital in Durban, South Africa. This hospital is a regional level facility and serves a predominantly semi–urban African population from where the participants were recruited. Normotensive (n = 95, age range: 18–46 years) and PE patients (n = 98, age range: 18–42 years) were enrolled into the study. Maternal venous blood samples were then taken. To maintain ethnographic and anthropometric consistency, all patients recruited were of African descent, resident in the same geographical location and of Zulu ethnicity. All patients were non-smokers, non-consumers of alcohol or recreational drugs, and all HIV infected patients were on HAART (tenofovir, emtricitabine, efavirenz) as per the National guidelines [7]. Calcium supplementation was administered routinely to all patients attending the clinic. Women with gestational hypertension, renal disease, diabetes mellitus, chronic hypertension and collagen vascular disease were excluded from this study. Preeclampsia was defined as a blood pressure ≥ 140mmHg systolic or ≥ to 90 mmHg diastolic on 2 occasions at least 4 h apart after 20 weeks of gestation in a woman with previously normal blood pressure [20]. All patients had proteinuria ≥ +1 on urine dipstick testing. Data on all patients was obtained from the institution’s maternity case records and laboratory data from the National Health Laboratory Services computerised database at the institution, and HIV was diagnosed on a rapid test kit. Weight was categorised as: normal weight (BMI: 18≤25), overweight (BMI: 25≤30) and obese (BMI: 30+).

### Cytokine quantification

The BD Cytometric Bead Array Human Th1/Th2/Th17 Cytokine kit (catalog no.560484) was used to measure the IL-2, TNF-α, IFN-γ and IL-6 levels in serum samples. Briefly, lyophilized standards were prepared by reconstitution and serial dilution (1:2–1:256) in assay diluent immediately before staining with Capture Beads and Phycoerythrin Detection Reagent. All serum samples were also diluted in assay diluent (1:4) before staining with Capture Beads and Phycoerythrin Detection Reagent. For the staining procedure, 50μL of each standard and unknown sample was added to appropriately labelled sample tubes followed by 50μL of the Human Th1/Th2/Th17 Phycoerythrin Detection Reagent and incubated (3 h, RT, protected from light). Following incubation 1 mL of Wash Buffer was added to each assay tube and centrifuged at 200 xg for 5 minutes. The supernatant from each assay tube was then carefully aspirated and 300 μL of Wash Buffer was added to each assay tube to resuspend the bead pellet. Flow cytometric data was acquired using the BD AccuriC6 Sampler counting 2,100 gated events. This ensures that the sample file contains approximately 300 events per Capture Bead. Data analysis was performed using the FCAP Array analysis software. All cytokines are represented as *p*g/mL as extrapolated from standard curves.

### Statistical analysis

Statistical analysis was done using SPSS^®^ version 22. Correlation between continuous variables was assessed using the Spearman rank correlation coefficient. The Wilcoxon rank-sum (Mann-Whitney) test was used to compare difference in sum of ranks (i.e. cytokine concentration) by dichotomous group, mainly between PE HIV− and PE HIV+. Comparisons of mean across 3 or more groups were done using the Kruskal–Wallis test. Non-parametric approaches were employed above as cytokine distributions were not normally distributed with evidence of asymmetry. The Pearson Chi-square (χ^2^) test was used to test association between group(s) and categorical explanatory variables. In the determination of significance, a p-value <0.05 was deemed statistically significant.

## Results

The clinical characteristics of participants are shown in Table 1. The average duration of HAART was 16.6 weeks in the control group and 14.5 weeks in the PE group. All women were in the third trimester of pregnancy and the mean gestational age was 36.5 weeks. There was a significant difference in the parity across all groups *(p = 0*.*006)* but not among the PE women *(p = 0*.*400)*.

**Table 1:** Clinical characteristics of participants.

Variable	PE HIV uninfected	PE HIV infected	Normo-tensive uninfected	Normo-tensive HIV infected	Total	p value	p value
Group	1	2	3	4	all	all	1 vs. 2
n	53	45	50	45	193		
**Age** (n, %)	53 (100)	44 (98)	50 (100)	45 (100)	192 (99)	0.002	0.003
mean ± SD	24.8 ± 5.3	28.7 ± 7.3	24.6 ± 6.4	28 ± 6.4			
range (yrs)	16–40	16–42	16–42	17–46			
**Parity** (n, %)							
0	26 (39.4)	18 (27.3)	17 (25.8)	5 (7.6)	66 (100)		
1–5	27 (22)	26 (21.1)	31 (25.2)	39 (31.7)	123 (100)		
> 5	0 (0)	1 (25)	2 (50)	1 (25)	4 (100)		
**GA** (wks)							
mean ± SD	35.8 ± 3.5	34.7 ± 4.6	38.2 ± 1.6	37.6 ± 2.7	36.5 ± 3.6	< 0.001	0.086
**CD4** (x10^6^/L)						0.399*	0
n, %	0	42 (93)	0	40 (89)			
mean ± SD		436 ± 181		432 ± 220			
**BMI** (n, %)	35 (66)	39 (87)	35 (70)	36 (80)	145 (76)		
mean ± SD	24.3 ± 13	39.4 ± 12.4	29.7 ± 12	30.1 ± 15.4	31 ± 7.4	0.391	0.229
**MOD** (n, %)						0	0
ELCS	9 (17)	9 (20)	25 (53)	27 (61)	70 (37)		
EMCS	32 (60)	23 (52)	13 (28)	11 (25)	79 (73)		
NVD	12 (23)	12 (27)	9 (19)	6 (14)	39 (21)		
Total	52 (100)	44 (98)	47 (94)	44 (98)	188 (98)		
**GA@sample** (wks)							
mean ± SD	35.6 ± 3.3	34.2 ± 4.5	38.2 ± 1.6	35.7 ± 2.7	189 ± 98	0.945	0.837
**EOPE** (n, %)	14 (26.4)	18 (40)			98 (100)		0.153
**Severe PE** (n, %)	18 (34)	21 (47)			98 (100)		0.2
**Proteinuria**	2+	2+			98 (100)		
**Systolic BP** (mm Hg)	157.1 ± 17.1	159 ± 14.2					
**Diastolic BP** (mm Hg)	101.9 ± 9.8	104.5 ± 9.8			98 (100)	0	0.304
**Alanine transaminase** (units/L)	26.3 ± 56.8	26.4 ± 53			89 (91)		0.993
**Aspartate transaminase** (units/L)	33.4 ± 34.7	64.2 ± 20.9			65 (66)		0.429
**γ-Glutamyl transferase** (units/L)	17.1 ± 14	26.9 ± 40.9			87 (89)		0.001
**Lactate dehydrogenase** (units/L)	630 ± 207.2	1052.7 ± 2047.6			43 (44)		0.39

Abbreviations: GA = gestational age, BMI = Body Mass Index, MOD = mode of delivery, ELCS = elective caesarean section, EMCS = emergency caesarean section, VD = vaginal delivery, SD = standard deviation PE = preeclampsia, n = total number (1): p = significance,

* = Grp 2 vs Grp 4

There was a significant difference in age across the groups, however there was little or no correlation between cytokine levels and age, negating age as a confounding factor; hence age was not factored into the cytokine comparison by group. The overall quantitative evaluation of cytokines is shown in Tables 2 and 3. Significant differences were found in many of the circulating cytokines investigated. In the control groups i.e. normotensive groups, significantly lower levels of IL-2 *(p = 0*.*010)*, TNF-α *(p = 0*.*045)*, and IL-6 *(p = 0*.*005)*, and a non-significantly lower level of IFN-γ *(p = 0*.*345)* in HIV infected women on HAART compared to uninfected controls.

**Table 2:** Quantitative evaluation of cytokines.

Cytokine	Statistics	PE + HIV+ {2}	PE - HIV- {3}	PE - HIV+ {4}
		vs.	vs.	vs.
		PE + HIV- {1}	PE + HIV- {1}	PE - HIV- {3}
**(1) IL-2** (pg/mL)	p-value	0.0008	0.9384	0.0104
	{group} [median]	{2} [268.7]	{3} [274.4]	{4} [257.6]
		{1} [273.2]	{1} [273.2]	{3} [274.4]
**(2) TNF-α** (pg/mL)	p-value	0.0001	0.2792	0.0453
	{group} [median]	{2} [172.2]	{3} [198.8]	{4} [174.8]
		{1} [185.2]	{1} [185.2]	{3} [198.8]
**(3) IFN-γ** (pg/mL)	p-value	0.0233	0.5534	0.3451
	{group} [median]	{2} [214.1]	{3} [217.1]	{4} [214.1]
		{1} [218.3]	{1} [218.3]	{3} [217.1]
**(4) IL-6** (pg/mL)	p-value	0.0865	0.7743	0.0051
	{group} [median]	{2} [139.7]	{3} [149.8]	{4} [133.0]
		{1} [147.0]	{1} [147.0]	{3} [149.8]

Abbreviations: PE = preeclampsia, HIV = Human Immunodeficiency Virus, IL-2 = interleukin 2, TNF-α = tumour necrosis factor alpha, IFN-γ = interferon gamma, i: Wilcoxon rank-sum (Mann-Whitney) test, IQR: interquartile range, vs = versus

**Table 3:** Comparative analysis of cytokine perturbations by groups.

	HIV+ vs. HIV-	PE vs. Normotensive	PE/HIV+ vs. PE/HIV-
**Group**	4 vs. 3	1 vs. 3	2 vs. 1
**Units**	Δ *median pg/mL* (p-value)		
**Cytokine**			
**IL-2**	-16.8 (0.010)*	-1.20 (0.938)	-4.5 (0.000)*
**TNF-α**	-24.0 (0.045)*	-13.6 (0.279)	-13.0 (0.000)*
**IFN-γ**	-3.0 (0.345)	+1.2 (0.553)	-4.2 (0.023)
**IL-6**	-16.8 (0.005)*	-2.8 (0.774)	-7.3 (0.086)

*(p< 0.05 is deemed statistically significant); Δ = difference in values; PE = preeclampsia

In the experimental group i.e. PE women, significantly lower levels of IL-2 *(p = 0*.*000)* and TNF-α *(p = 0*.*000)* and non-significantly lower levels in IFN-γ *(p = 0*.*023)* and IL-6 *(p = 0*.*086)* were observed in HIV infected women on HAART compared with uninfected women. No significant differences were observed in uninfected PE women when compared with uninfected normotensive women.

## Discussion

Our study demonstrates that both normotensive and PE HIV-infected women on HAART display lower cytokine levels than both PE and normotensive uninfected pregnant women. The data presented shows significantly lower levels of pro-inflammatory cytokines IL-2, TNF-α, and IL-6, and non-significantly lower IFN-γ among HIV positive normotensive women on HAART compared with uninfected normotensive pregnant women (Table 3). A similar profile was observed in PE women, with significantly lower IL-2 and TNF-α levels and non-significant lower IFN-γ and IL-6 (Table 2) in HIV infected PE women on HAART relative to uninfected PE women. This is suggestive of HIV in combination with HAART having suppressive effects on these inflammatory cytokines during pregnancy in both uncomplicated and PE pregnancies. Similar findings were observed in a local study among Black African participants receiving HAART, although this study involved non-pregnant participants [21].

Cytokines have been implicated as potential mediators in PE, where endothelial dysfunction is considered the hallmark of the syndrome [22]. Cytokines may also be involved with abnormal inflammatory responses caused by syncytiotrophoblast molecules (STBM) shed into maternal blood in PE [13]. The alteration between pro-inflammatory/regulatory responses does not occur in PE, or may be reverted in very early stages of the disease, leading to a pro-inflammatory state [23].

In our study, we did not find significant differences in IL-2, TNF-α, IFN-γ or IL-6 in PE women compared to normotensive women. Our findings do not reflect the pro-inflammatory environment in PE shown in other studies [24–26], however differences in study designs exist in relation to sample population, pregnancy status, and sampling techniques.

T cells are cytokine producing cells that are infected by HIV via CD4^+^ receptors present on the cell surface [27]. Studies conducted earlier have revealed that HIV-infected individuals have a weaker immune system and the inability of CD4^+^ T cells to proliferate, due to the decrease in the levels of IL-12 [28]. As a result, decreases in IL-2 and IFN-γ occur, leading to immunosuppressive effects and opportunistic infections, a marker of advanced disease. In a recent study of the cytokine milieu in untreated HIV infection, pro-inflammatory cytokines, IL-2, IL-12, and IFN-γ were shown to be significantly decreased. CD4 cell counts are an important biomarker for HIV progression, and are lower in HIV participants versus healthy participants [27]. In our study, we did not find a significant difference between the CD4^+^ counts in normotensive HIV infected women and those with PE, however, both groups were on HAART in terms of existing guidelines [7].

Due to HIV having immune—depressive effects, an association between PE and HIV has been suggested [29]. HAART suppresses HIV viremia, increases CD4^+^ cell counts, and is suggested to counteract the Th1 to Th2 shift in the disease progression of HIV [19]. The use of HAART in pregnancy provides significant benefits in delaying HIV disease progression and reducing the risk of mother-to-child-transmission, and has been integrated into policy [7].

Our data suggests that immune reconstitution by HAART in these conditions includes alterations in pro-inflammatory cytokines. Although some clinical data is associated with a lower rate of PE among HIV positive women who receive HAART [30], a further prospective cohort study with sequential sampling is necessary to determine the clinical association. Currently there is a paucity of data on cytokine mediated immune reconstitution effects associated with HAART in pregnancy, possibly due to differences in drug regimens, patient profiles, study settings and rapidly evolving therapeutic regimens.

Our study was limited by the lack of knowledge on the precise duration of HAART and detailed knowledge of drug adherence. Moreover, pregnancy is inherently immunogenic and is associated with longitudinal variation, posing further challenges to accurately contextualise changes. In our study, we did not include patients with untreated HIV infection, which is unethical under the current guidelines. In addition, we included patients that had proteinuria ≥ +1 on urine dipstick testing which may have included patients without significant proteinuria (≥ 300mg). The present study was conducted among African women, and our results may therefore differ in comparison to other HIV-infected populations. In the context of racial variation on cytokine responses, African–Americans have been shown to have higher baseline levels of inflammatory cytokines [31]. Differences in sample sizes, patient selection and techniques used, further contribute to heterogeneity among studies relating to PE and HIV. Furthermore, functional pleiotropy and redundancy are characteristic features of cytokines, and may show overlapping activities depending on the type and developmental state of the target cells involved [32].

## Conclusion

The effects of HAART, in conjunction with HIV, during pregnancy include alterations in pro-inflammatory cytokines IL-2, TNF-α, IFN-γ and IL-6. We observed consistently lower levels of IL-2 and TNF-α in HIV-infected pregnant women on HAART, regardless of PE or normotensive status; suggesting HIV/HAART has an inhibitory effect on these proinflammatory cytokines during pregnancy. Under normotensive conditions, IL-6 was significantly lower in HIV infected women on HAART. These findings highlight the need for further investigation on the immune reconstitution effects of HAART during pregnancy, and the potential of immune modulation therapy for the management of PE, where treatment still remains empiric.
